# Improving clinical judgment by simulation: a randomized trial and validation of the Lasater clinical judgment rubric in Chinese

**DOI:** 10.1186/s12909-019-1454-9

**Published:** 2019-01-14

**Authors:** Fen Yang, Yuncui Wang, Chongming Yang, M Huifang Zhou, Jing Shu, Bei Fu, Hui Hu

**Affiliations:** 1 School of Nursing, Hubei University of Chinese Medicine, Wuhan, China; 2 Research Support Center, Brigham Young University, Provo, UT USA

**Keywords:** Chinese Lasater clinical judgment rubric, Standardized patients, Simulation education

## Abstract

**Background:**

The development and assessment of clinical judgment ability are essential in nursing education. The Lasater Clinical Judgment Rubric (LCJR) was shown to be valid in evaluating nursing students’ learning outcomes and skills in western cultures but has not been validated in mainland China. This study aimed to compare a simulation-teaching model with a traditional teaching method in enhancing the clinical judgment ability of nursing undergraduate students and to validate the Chinese version of the Lasater Clinical Judgment Rubric (C-LCJR).

**Methods:**

Four classes of nursing students (*n* = 157) at Hubei University of Chinese Medicine, China, were randomly assigned to two control and two experimental classes. The experimental classes were taught using simulation teaching with standardized patients, while the control classes were taught using traditional teaching methods. At the end of the experiment, students in both kinds of classes evaluated their clinical judgment using the C-LCJR. Teachers also rated the students but without knowing who had received the simulation teaching. Confirmatory factor analysis and a Multiple Indicators Multiple Causes (MIMIC) model with Bayesian estimation was fit to the rating data to investigate measurement properties and experimental effects.

**Results:**

Compared to the control classes, students in the experimental classes performed better in all subdomains of C-LCJR (noticing, interpreting, responding, and reflecting). The measurement properties of the C-LCJR were found to be satisfactory with high factor loadings and reliabilities and no bias from age, gender, and raters.

**Conclusions:**

The simulation teaching model is more effective than the traditional (non-simulation-based) teaching method in improving clinical judgment of Chinese nursing students. The C-LCJR is a valid and reliable instrument for measuring clinical judgment in nursing students in China.

## Background

Clinical judgment is imperative for professional nurses. Tanner defined clinical judgment as “an interpretation or conclusion about a patient’s needs, concerns, or health problems and/or the decision to take action (or not), use or modify standard approaches, or improvise new ones as deemed appropriate by the patient’s response” [1]. The clinical judgment model includes four phases: noticing, interpreting, responding, and reflecting [1]. Effective clinical judgment is essential to ensure patient safety and quality nursing care [2]. On the contrary, its absence increases the possibility of adverse events [3–5]. In line with this, acquiring clinical judgment is a key teaching objective of nursing curricula. The challenge for nursing educators is to implement teaching techniques that improve students’ clinical judgment and to effectively navigate how to evaluate such techniques validly and reliably.

The development of clinical judgment in nursing students entails simulation teaching. As nursing students have not yet acquired sufficient judgment and skills, allowing them to practice in real clinical settings can cause tremendous concerns from patients [6]. Benner [7] found that it often takes one or 2 years of clinical experience for a novice nurse to become an expert. To help nursing students adapt to the clinic as soon as possible, simulation teaching has become a supplemental strategy to improve and assess their clinical judgment [8].

Simulations have been identified as an innovative approach to education that attempts to imitate important aspects of clinical cases [9]. Kaddoura et al. [10] maintained that simulation is a potent teaching and learning method for developing clinical judgment among nursing students. Furthermore, students can develop critical thinking skills through decision-making and problem-solving and maintain a safe training without worrying about injuring patients [11]. A recent study showed that simulation safely replaced up to 50% of clinical education without reducing learning or ability and should be used more in nursing education [12]. A simulation with standardized patients (SPs) provides opportunities for students to apply their skills on persons trained to impersonate the characteristics of a real patient in a safe and controlled environment prior to taking care of real patients in clinic [13]. Teaching with SPs has many advantages, including increasing students’ communication skills with patients and with teammates [14], improving clinical reasoning [15], and reducing students’ anxiety and stress and increasing their self-efficacy and study motivation [16]. Therefore, students can develop critical-thinking skills through decision-making and problem-solving so as to maintain a safe training environment without worrying about injuring patients.

The Lasater Clinical Judgment Rubric (LCJR) was developed to evaluate simulation experience based on Benner’s seminal novice-to-expert model [1, 17] and Tanner’s clinical judgment model [1]. The LCJR evaluates expected student performance according to Tanner’s four phases of clinical judgment (noticing, interpreting, responding, and reflecting) at various levels. It has been found to be an effective and reliable standard to assess the cognitive and emotional aspects of the clinical judgment of nursing students in simulation exercises [18]. The LCJR allows instructors to discover the potential of each student and provide timely feedback. It can promote communication through clear feedback of the results in a clinical or simulation environment [19]. It can also be used by evaluators as an observation tool [19, 20] or by students as a self-assessment exercise [21]. Several studies have used the LCJR to compare nursing students’ self-assessments with teachers’ assessments and to evaluate the clinical judgment of nursing students through repeated measurement designs [22, 23]. Some studies have shown high internal consistency (Cronbach alpha = 0.80–0.97) [24–26], but have provided no information on other essential psychometric properties like dimensionality and factor loadings.

The LCJR has been used in the United States [18, 25–28] and has been translated into Sweden [29], Korean [30], Lebanon [31], Spanish [32] and Dutch [33], and validated in the corresponding countries. To our knowledge, no Chinese version was available at the time this report was prepared. Therefore, one aim of this study was to establish a Chinese culturally adapted and validated version of the LCJR and examine its validity with empirical data of Chinese nursing students receiving simulation teaching. Another aim was to compare simulation teaching with traditional teaching methods of nursing students in the Chinese culture. Our hypothesis is that simulation teaching with standardized patients improves clinical judgment more effectively than traditional (non-simulation-based) teaching methods.

## Methods

### Participants

**Fig. 1:**
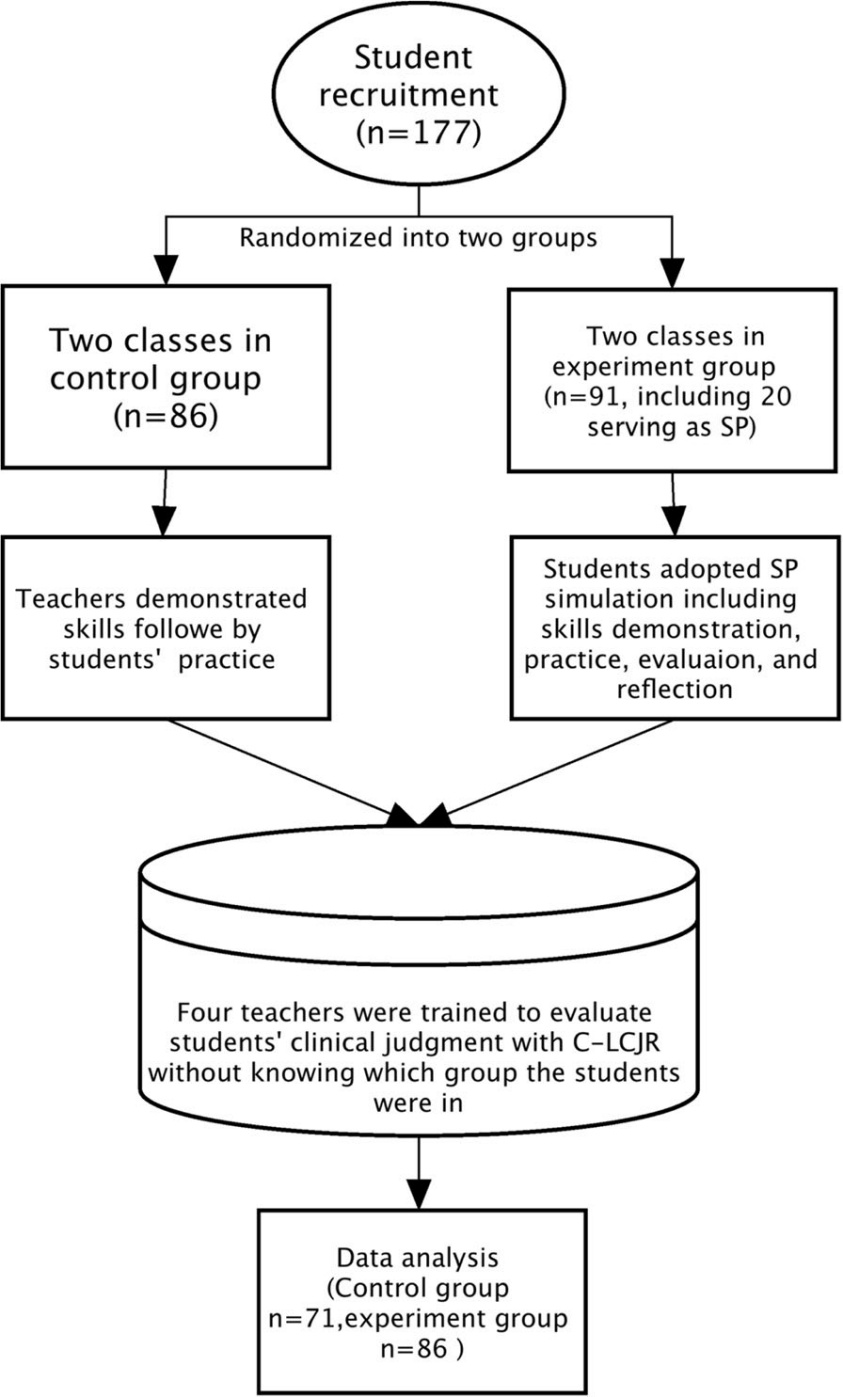
Flow-chart for this research progress

A total of 177 sophomore nursing students (March–June 2015) were recruited from four classes in the college of nursing at Hubei University of Chinese Medicine in Wuhan, China. We used the grasping and smashing method to divide 4 classes into 2 groups. The 4 classes were randomized into 2 control (*n* = 86) and 2 experimental classes (*n* = 91, including 20 students serving as SPs). The final sample for the statistical modeling had 71 (45.2%) in the experimental group and 86 (54.8%) in the control group. The flowchart for the research is shown in Fig. 1. Female students comprised 89.8% and male 10.2% of the sample. The average age of the sample was 19.7 years (SD: 0.88). All the participants had no prior clinical practice or simulation experience.

A priori sample size was not determined for the study because the appropriate sample size depends on many parameters and their sizes, including factor loadings of the measurement model, covariate effects, and the main experimental effects [34]. Inappropriate specifications could lead to different estimates of the desirable sample sizes. Therefore, a power analysis was conducted to estimate the power of each parameter of the model at the current sample size.

### Measurement

The LCJR was used to measure students’ clinical judgment [35]. The rubric has 4 dimensions (noticing, interpreting, responding, and reflecting) and a total of 11 items on which a participant is evaluated as beginning (1 point), developing (2 points), accomplished (3 points), or exemplary (4 points. The overall score may range from 11 to 44. Higher scores indicate better clinical judgment [36]. In brief, the noticing dimension emphasizes the ability to gather and recognize information. The interpreting dimension involves prioritizing relevant information and interpreting it to explain a patient’s condition. The responding dimension focuses on style habits, communication skills, intervention/flexibility, and the use of nursing skills. The reflecting dimension involves self-evaluating and commitment to improvement. For details on the four dimensions, readers may refer to Miraglia and Asselin [36].

To construct the Chinese version of the LCJR, the original English edition of the LCJR was translated into Chinese by researchers after obtaining permission from its author. A group of experts with experience in education simulation revised the first Chinese translation according to various suggestions and enhanced its understandability and acceptability to Chinese nursing teachers and students. The final draft of the Chinese LCJR (C-LCJR) was translated back into English and compared with the original version. After slight modifications for semantic differences, the final version of the C-LCJR was used in this study.

### Procedure

Twenty students were selected from the experiment classes to serve as SPs. These students were selected based on their talents, enthusiasm, and commitment to complete the tasks. The simulation teaching was composed of 5 major scenarios, including care for patients with a cold, cervical spine pain, dysmenorrhea, heat stroke, and insomnia. The SPs were trained for 3 h for each scenario and spent at least 3 h per week practicing until passing the SPs assessment.

The experimental classes adopted SP simulation teaching in groups of 5 or 6 students. Each simulation lasted 1 hour and included a demonstration of skills, practice, self-evaluation, teachers’ evaluations, and reflection. The simulation course proceeded with a pre-learning activity, simulation and practice, and writing in reflection diaries. Each student participated in 3 simulation sessions of different designs. The participating teachers were trained to ensure the consistency of the simulation teaching. In contrast, students in the control classes viewed demonstrations of operations and then practiced them, according to traditional teaching methods.

After the simulation teaching (June 2015), students in both the control and experimental classes participated in one of the simulation scenarios that was recreated with SPs and that they had not practiced before. Students then rated their own clinical judgment using the C-LCJR. The grouping of students for the final assessment was carried out by another teacher who was the data analyst and four teachers in the assessment who were blinded and did not know the grouping information of the students in advance and were trained to use the C-LCJR to rate the students in these recreated scenarios. When the entire evaluation process was over, data analysts organized and analyzed the data. Other information (age, gender, and class) was collected with questionnaires.

### Data analysis

To maximize the use of the rating information from both students and teachers, the measurement properties of the C-LCJR were first examined with a 2-level (individual rater and two raters) Confirmatory Factor Analysis (CFA), in which the constructs of the original LCJR were specified as latent variables and ratings of the items as categorical indicators. Such treatment specifies a probit modeling of the relations between the latent variables and their observed indicators. This latent variable modeling partitions out measurement errors and yields more accurate estimates of the experimental effects in subsequent modeling than does traditional analysis of variance of sum scores [37]. Bayesian estimation was adopted to accommodate the small sample size and probabilistic interpretation of the estimates. In Bayesian analysis, each parameter has a distribution like a variable with a mean and median instead of a single constant. As the distribution may not be normally distributed, preferred reporting is usually the median, below and above which half of the estimates fall. Model fit was indicated by the posterior predictive *p*-values (*ppp*), with *ppp* > .05 implying an acceptable fit [38]. The 95% credibility interval of an estimate suggests that the 95% probability for the estimate is within the lower and upper limits, as listed in a bracket in the Results section. Readers interested in more practical applications may refer to Muthén and Asparouhov [39].

Reliability (ω) of each subscale was calculated instead of the traditional reliability measure (Cronbach’s alpha) that assumes equal factor loadings of continuous variables, which made it inappropriate for these measures [40].

The experimental effects were examined with a 2-level Multiple Indicators Multiple Causes (MIMIC) model, which specified the 4 dimensions of the C- LCJR as the endogenous variables and a dummy-coded grouping variable (experimental = 1, control = 0) as the exogenous, while controlling for other covariates to further balance any differences in the students between the experiment and control classes. The following covariates were initially included in the model: class, gender, age, group, and evaluator. Female gender, students’ self-evaluation, and control group were used as the reference group in analyses. Measurement invariance was also explored by estimating the effects of the covariates on the indicators of the subdomains [41]. Figure 1 depicts the model simplified to retain only the significant exogenous effects. All analyses and modeling were carried out with the latent variable modeling program Mplus (v8.1).

## Results

### Experimental and covariate effects

**Fig. 2:**
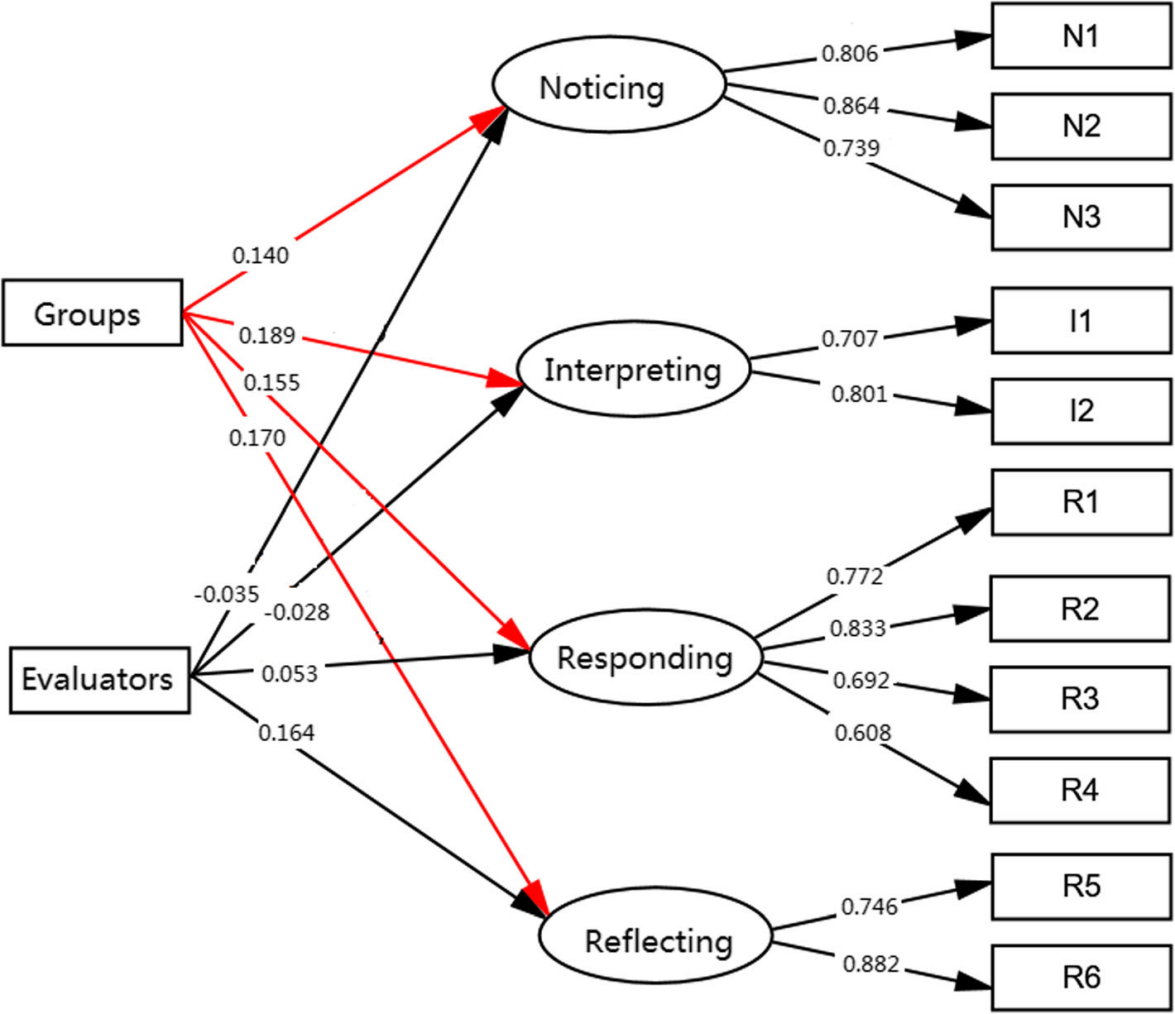
Multiple Indicators Multiple Causes Model of LCJR. Showed the differences between self- and teacher rating on the four dimension of the C-LCJR Groups = intervention and control group; Evaluators = self-rating and assessors

The final model that estimated the experimental effects (Fig. 2) fit the data well, with *ppp* = .36. Students in the experimental group performed better than those in the control group in all subdomains of C-LCJR, as indicated by the significant experimental effects, respectively, on noticing (γ = 0.14, *p* < .05, CI [0.02, 0.29]), interpreting (γ = 0.19, *p* < .05, CI [0.03, 0.36]), responding (γ = 0.16, *p* < .05, CI [0.01, 0.31]), and reflecting (γ = 0.17, *p* < .05, CI [0.02, 0.33]). In addition, teachers rated higher than students on the reflecting domain as indicated by the significant rater effect (γ = 0.17, *p* < .05, CI [0.02, 0.33]). The minimum power for these group differences (as group effect) is .77 but is over .90 for the factor loadings at the sample size of this study.

Gender, class, and age did not have any significant effects on any subdomains or their indicators in the model, implying that the experimental and control classes were well-balanced or the experimental effects were not biased by these variables. These covariates were excluded from the final model for parsimony.

### Measurement properties of C-LCJR

The factor structure of the C-LCJR and other influencing factors were tested using the MIMIC model of CFA and Bayesian effects estimation. The CFA showed that the model fit the data well (*ppp* = 0.37). The posterior median estimates of the standardized factor loadings are listed in Table 1. All factor loadings were statistically different from zero (*p* < .01). The inter-correlations of the 4 domains ranged from .45 to .83. Although noticing and interpreting are highly correlated at .83, model comparisons (3 factors vs. 4 factors) showed that the 4 domains are distinct, as combining noticing and interpreting into one factor significantly worsens the model fit. The reliabilities of the C-LCJR subdomains listed in Table 1 are all above .70, the conventional standard for a desirable scale.

**Table 1:** Bayesian estimates of reliability and factor loadings [95% credibility intervals]

Items	Reliability	Factor Loadings
Noticing	0.84 [.72, .92]	
N1 Focused observation		0.78 [0.67, 0.88]
N2 Recognizing deviations		0.87 [0.78, 0.94]
N3 Information seeking		0.74 [0.60, 0.84]
Interpreting	0.71 [.56, .82]	
I1 Prioritizing data		0.67 [0.54, 0.78]
I2 Making sense of data		0.82 [0.70, 0.90]
Responding	0.81 [.67, .89]	
R1 Calm, confident manner		0.75 [0.63, 0.85]
R2 Clear communication		0.82 [0.71, 0.90]
R3 Well-planned intervention		0.68 [0.53, 0.80]
R4 Being skillful		0.60 [0.43, 0.74]
Reflecting	0.79 [.64, 88]	
R5 Evaluation		0.73 [0.61, 0.83]
R6 Commitment to improvement		0.88 [0.76, 0.95]

Note: These estimates are at within-individual level instead of between-raters’ level

## Discussion

This study aimed to examine the experimental effect of simulation teaching with SPs on nursing students and to evaluate the measurement properties of the C-LCJR in China. The results showed that simulation teaching with SPs outperformed traditional teaching methods in facilitating the development of clinical judgment in Chinese nursing students, as is consistent with previous findings [10]. Using SPs in clinical simulation enabled nursing students to employ the theoretical information and skills presented in the classroom and in their clinical practice and specifically enhanced their sense of clinical judgment [23].

Our results also showed no significant effects of age, gender, and classes on the subdomains of clinical judgment, implying that randomization at the class level did not result in any bias by these known variables, as opposed to a randomization at the participants’ level. The results of other covariates were similar with one study [42]. Another study by Vreugdenhil and Spek [33] found that the score differences were not significant between students’ self-ratings and nurse educators’ assessments. Lasater [35], however, believed that clinical judgment could be influenced by individual characteristics. Future studies may collect personality or intelligence data about the instructors, SPs, and students to examine any potential main and moderating effects.

The results also showed that the C-LCJR had satisfactorily high factor loadings and reliabilities (ω) and no bias from age, gender, or raters. The high reliabilities are consistent with the findings of Victor-Chmil et al. [18] and Luiking et al. [43] that nursing practices in the US and in the Netherlands show more similarities than differences. Reliability from different LCJR studies ranged from .57 to 1.00 [25, 43]. These findings suggest that C-LCJR is applicable to nursing students in China.

There are several limitations of our study. First, the participants of this study were selected from one school, which might limit the generalizability to students of other majors or schools. Second, the participants did not have any prior clinical experience, there is challenge to develop SPs with standardized clinical experience, so the experimental effects may be confounded with a floor effect. Future studies may be designed to control for prior levels of clinical judgment to examine experimental effects of varied simulation teaching. Third, no sample size calculations were made, and no baseline measurements were taken. This may affect the quality and intentional analysis of the article.

## Conclusions

In summary, the C-LCJR had satisfactory measurement properties for Chinese nursing students and thus can be used by educators to evaluate nursing students’ clinical judgment and can also be used by students to assess themselves. Simulation teaching with SPs is an effective method that outperforms traditional teaching methods in helping nursing students develop clinical judgment.

## Abbreviations

C-LCJR: Chinese Lasater Clinical Judgment Rubric
LCJR: Lasater Clinical Judgment Rubric
SP: Standardized patients
